# Design and Evaluation of a Proxy-Based Monitoring System for OpenFlow Networks

**DOI:** 10.1155/2016/6513649

**Published:** 2016-02-23

**Authors:** Yoshiaki Taniguchi, Hiroaki Tsutsumi, Nobukazu Iguchi, Kenzi Watanabe

**Affiliations:** ^1^Faculty of Science and Engineering, Kindai University, 3-4-1 Kowakae, Higashi-Osaka 577-8502, Japan; ^2^Graduate School of Science and Technology, Kindai University, 3-4-1 Kowakae, Higashi-Osaka 577-8502, Japan; ^3^Graduate School of Education, Hiroshima University, 1-1-1 Kagamiyama, Higashi-Hiroshima 739-8524, Japan

## Abstract

Software-Defined Networking (SDN) has attracted attention along with the popularization of cloud environment and server virtualization. In SDN, the control plane and the data plane are decoupled so that the logical topology and routing control can be configured dynamically depending on network conditions. To obtain network conditions precisely, a network monitoring mechanism is necessary. In this paper, we focus on OpenFlow which is a core technology to realize SDN. We propose, design, implement, and evaluate a network monitoring system for OpenFlow networks. Our proposed system acts as a proxy between an OpenFlow controller and OpenFlow switches. Through experimental evaluations, we confirm that our proposed system can capture packets and monitor traffic information depending on administrator's configuration. In addition, we show that our proposed system does not influence significant performance degradation to overall network performance.

## 1. Introduction

In recent years, the demand for networking has changed along with the popularization of cloud environment and server virtualization. For example, virtual machines are easily added or moved among servers in a data center. Along with the variation of virtual machines, it is necessary to configure network devices appropriately. In traditional networks, individual network device is managed manually by an administrator. Therefore, it takes high cost to change the configuration of networks. In addition, in traditional network devices, only limited control functions provided by the vendor are available for use.

Recently, Software-Defined Networking (SDN) has attracted attention from many researchers and developers [1–5]. In SDN, the control plane and the data plane are decoupled so that the logical topology and routing control can be configured dynamically depending on network conditions. In addition, the control plane can be developed by users or administrators so that the network can be easily controlled depending on administrator's configurations.

To obtain network conditions precisely for dynamic control of SDN, a network monitoring mechanism is necessary. In addition, if we obtain traffic information or capture entire packets in a specific area in a network, these information pieces can be used for protecting unusual traffic or packets as well as for transferring malicious packets to a honey pot. Until now, there have been some network monitoring systems [6–8]. However, these monitoring systems do not support flow-level monitoring, packet capturing capability or can be operated only in a specific network.

**Figure 1:**
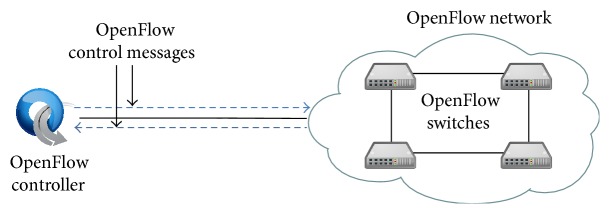
The architecture of OpenFlow network.

**Figure 2:**
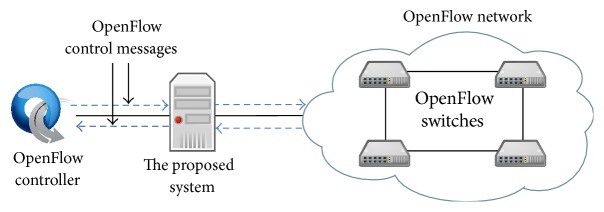
The architecture of OpenFlow network with our proposed monitoring system.

In this paper, we propose, design, implement, and evaluate a network monitoring system focusing on OpenFlow [9] which is a core technology to realize SDN. An OpenFlow network consists of an OpenFlow controller and OpenFlow switches as shown in Figure 1. The OpenFlow controller is used as control plane and the OpenFlow switches are used as data plane. Our proposed system acts as a proxy between an OpenFlow controller and OpenFlow switches as shown in Figure 2. Our proposed system can capture packets and monitor traffic information depending on configuration. The configuration is set by an administrator through a web interface or by other systems through REST API. In addition, our proposed system works in a network which is compliant with OpenFlow specification and does not depend on the vendor of controller and switches. In this paper, we evaluate our proposed system through experimental evaluations.

The rest of this paper is organized as follows. In Section 2, we explain related work. Next, we propose the network monitoring system in Section 3. Then, we evaluate the proposed system in Section 4. Finally, we conclude this paper with an outlook on future work in Section 5.

## 2. Related Work

In this section, we briefly explain OpenFlow networks and traditional monitoring systems.

### 2.1. OpenFlow Networks

SDN has attracted a lot of attention since the OpenFlow project was started at Stanford University. Until now, there have been a lot of researches for OpenFlow networks [10–13].

An OpenFlow network consists of an OpenFlow controller and OpenFlow switches as shown in Figure 1. The OpenFlow controller is used for the control plane. There are some open source development environments for OpenFlow controller such as POX [14], Trema [15], Ryu [16], and Floodlight [17]. On the other hand, OpenFlow switches are used for data plane. For each OpenFlow switch,* Datapath ID* is assigned for identification. In addition, an OpenFlow switch has a table called* flow table* to forward packets. The flow table consists of* flow entries*, each of which consists of match fields, counters, priorities, instructions, and so on. A match field is used for matching between incoming packet and flow entry. Counters are used to obtain statistics of packets.

When an OpenFlow switch receives a packet, it first checks its flow table. If there is a flow entry which matches with the packet header information, the switch processes the packet according to the instruction contained at the flow entry. If there are some flow entries which match with the packet header information, the flow entry with the highest priority is chosen. Otherwise, if there is no flow entry, the OpenFlow switch sends a Packet-In message to the OpenFlow controller. When the OpenFlow controller receives the Packet-In message from the switch, it determines how to process the packets and notices to the OpenFlow switch using a Flow-Mod message. Therefore, the whole behavior of OpenFlow network is determined by the OpenFlow controller.

### 2.2. Traditional Monitoring Systems

Until now, there have been many monitoring systems [6–8] for network and network devices.  Table 1 summarizes the comparison of traditional monitoring systems and our proposed system.

The Simple Network Management Protocol (SNMP) [6] is the most typical protocol for monitoring traditional networks. In SNMP, there are two components: an SNMP agent and an SNMP manager. An SNMP agent runs on managed device and it has Management Information Bases (MIBs) for maintaining device information. An SNMP manager is management software and it can obtain information on managed devices through communication with the SNMP agent. In SNMP, although interface-level traffic monitoring is possible by measuring traffic for each interface, flow-level monitoring is not possible. On the other hand, in our proposed system, not only interface-level monitoring but also flow-level monitoring is possible.

NetFlow [7] is a protocol for monitoring traffic information developed at the Cisco Systems. Although flow-level monitoring is possible by using NetFlow, NetFlow does not support packet capture capability and upper layer information can not be obtained. On the other hand, our proposed system is capable of capturing packets. Our proposed system can capture packets in a specific area of network and upper layer information can be obtained.

Big tap [8] is an SDN monitoring tool developed at the Big Switch Networks. By using Big tap, port mirroring of an interface is possible and the traffic can be forwarded to applications for further analysis. In addition, Big tap supports packet filtering based on packet header information. However, Big tap is only available on the Big Network Controller, and it is not available at other controllers. On the other hand, since our proposed system acts as a proxy, our proposed system is available at any OpenFlow network compliant with OpenFlow specification.

## 3. Proposed System

In this section, we propose a monitoring system for OpenFlow networks.

### 3.1. Overview

**Figure 3:**
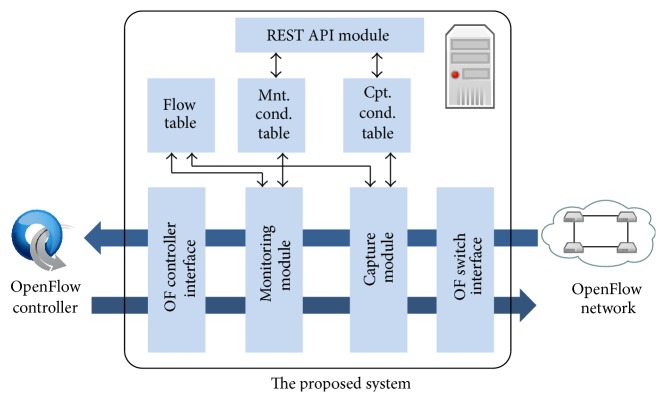
The modules constitution of our proposed system.

Figure 3 shows modules that constitute our proposed system. Our proposed system acts as a proxy between an OpenFlow controller and OpenFlow switches to capture packets and to monitor traffic information. When an OpenFlow switch sends a message to our proposed system through the* OF switch interface*, our proposed system forwards the message to the OpenFlow controller using* OF controller interface*. On the other hand, when the OpenFlow controller sends a message to our proposed system, our proposed system forwards the message to the corresponding OpenFlow switch. Therefore, all messages between the OpenFlow controller and OpenFlow switches can be monitored at our proposed system.

In addition, our proposed system can capture packets and monitor traffic using* capture module* and* monitoring module*, respectively. The configurations are maintained using* capture condition table* and* monitoring condition table*. These configurations are set by an administrator through a web interface or by other systems through* REST API module*. In the following sections, we explain the details of the capture module and the monitoring module.

### 3.2. Capture Module

**Figure 4:**
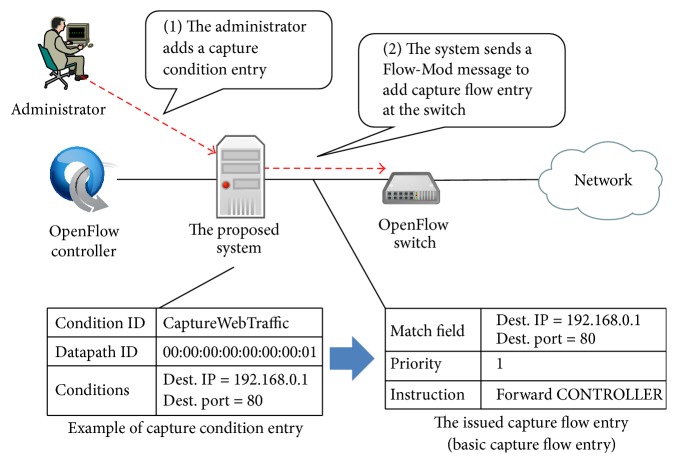
The basic behavior of capture module in the case of capture condition entry addition.

The capture module is used for packet capturing. To maintain configuration for capturing packets, the capture module refers to the capture condition table. Each condition entry in the capture condition table consists of condition ID, Datapath ID, and conditions. The constitution of conditions is shown in Table 2, and an example of capture condition entry is illustrated in Figure 4. In addition, the capture module refers to the flow table, which maintains flow entries issued from the OpenFlow controller to an OpenFlow switch. In the following, we explain the details of behavior of packet capture module.

#### 3.2.1. Behavior against Addition of Capture Condition Entry

When a capture condition entry is added, the capture module generates a Flow-Mod message to add new flow entry for packet capturing at the OpenFlow switch. Each field in the Flow-Mod message is determined as follows. The match field is set to the condition in the capture condition entry. The instruction is set to Forward CONTROLLER to forward received packets to our proposed system at the OpenFlow switch. The priority is set to the lowest value 1. Then, the capture module sends the generated Flow-Mod message to the corresponding OpenFlow switches. An example of capture condition entry and generated flow entry is shown in the bottom of Figure 4. In the following, we denote the added flow entry for packet capturing at the OpenFlow switch as* capture flow entry*. In particular, we denote the capture flow entry with the lowest priority value as* basic capture flow entry*.

At an OpenFlow switch, if there is intersection between a match field in an existing flow entry and a match field in the basic capture flow entry, the capture flow entry does not work since the priority is set to the lowest value. Therefore, in this case, the capture module generates additional Flow-Mod message as follows.

**Figure 5:**
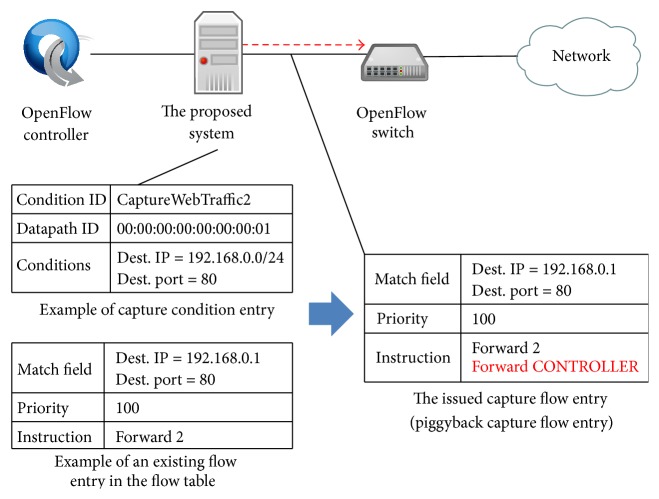
Additional Flow-Mod message for piggyback capture flow entry.

**Figure 6:**
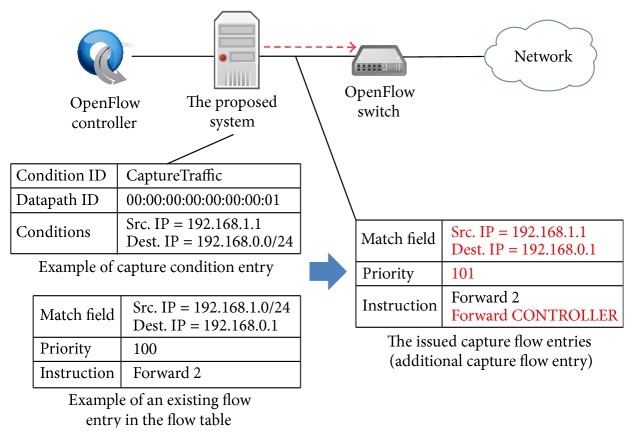
Additional Flow-Mod message for additional capture flow entry.

- If a match field in an existing flow entry is a part of conditions in the capture condition entry, the capture module generates Flow-Mod message based on the existing flow entry by adding Forward CONTROLLER instruction as shown in Figure 5. We denote the capture flow entry made by this message as* piggyback capture flow entry*.
- On the other hand, if a part of match field in an existing flow entry matches with a part of conditions in the capture condition entry, the capture module generates a Flow-Mod message as shown in Figure 6. In the Flow-Mod message, the match field is set to the intersection of the existing match field and the conditions in the capture condition entry. The instruction is set based on the instruction in the existing flow entry and Forward CONTROLLER instruction. The priority is set based on the existing flow entry by adding one. We denote the capture flow entry made by this message as* additional capture flow entry*.

Then, the capture module sends the generated additional Flow-Mod message to the corresponding OpenFlow switches.

Here, we note that the OFPFF_SEND_FLOW_REM flag is set to be true in all Flow-Mod messages sent from our proposed system. When a flow entry with this flag is deleted at a switch, Flow-Removed message is sent to the controller.

#### 3.2.2. Behavior of Packet Capturing

**Figure 7:**
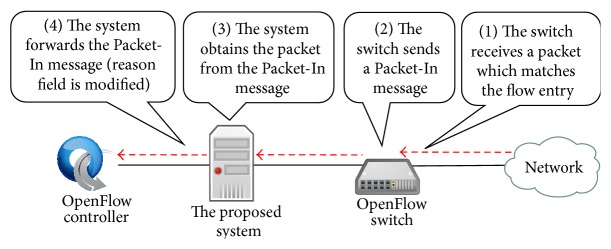
The behavior of capture module in the case of Packet-In message reception from the OpenFlow switch.

When an OpenFlow switch receives a packet which matches the capture flow entry, it sends the received packet using Packet-In message to our proposed system as shown in Figure 7. When the capture module in our proposed system receives the Packet-In message, it obtains the original packet from the message. Therefore, packet capturing can be accomplished in our proposed system at this stage.

Here, if the packet header information in the received Packet-In message does not match any flow entries in the flow table in our proposed system, it means that the message is sent by basic capture flow entry. In this case, the reason field in the received Packet-In message is set as OFPR_ACTION, which means that the message is sent by capture flow entry. However, the capture flow entry is added not by the OpenFlow controller but by the capture module in our proposed system. Therefore, the capture module modifies the reason filed to OFPR_NO_MATCH, which means that the message is sent due to table miss. Then, the capture module sends the modified Packet-In message to the OpenFlow controller as shown in Figure 7. On the other hand, if the packet header information in the received Packet-In message matches with a flow entry in the flow table, the capture module discards the message and does not forward it to the OpenFlow controller.

#### 3.2.3. Behavior against Reception of Flow-Mod Message

When our proposed system receives a Flow-Mod message from OpenFlow controller, the capture module acts depending on the relationships between flow entry in the Flow-Mod message and capture condition entry as follows.

- If a match field in the Flow-Mod message is a part of conditions in a capture condition entry, the capture module adds Forward CONTROLLER instruction. The capture flow entry made by this message corresponds to piggyback capture flow entry.
- On the other hand, if a part of match field in the Flow-Mod message matches with a part of conditions in a capture condition entry, the capture module generates additional Flow-Mod message. In the additional message, the match field is set to the intersection of the match field in the message and the conditions in the capture condition entry. The instruction field is set based on the flow entry and Forward CONTROLLER instruction, and the priority field is set based on the flow entry by adding one. The capture flow entry made by this additional message corresponds to additional capture flow entry.

**Figure 8:**
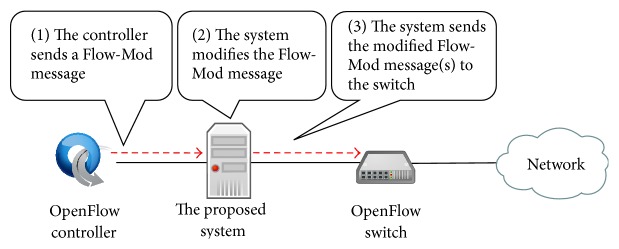
The behavior of capture module in the case of Flow-Mod message reception from the OpenFlow controller.

Then, the capture module forwards the message to the corresponding OpenFlow switch as shown in Figure 8.

#### 3.2.4. Behavior against Reception of Flow-Removed Message

When a flow entry is deleted at the OpenFlow switch due to some reason such as timeout, Flow-Removed message is sent to our proposed system. If a flow entry related to an additional capture entry is deleted at the switch, the capture module deletes the additional capture entry at the OpenFlow switch by sending a Flow-Mod message.

#### 3.2.5. Behavior against Removal of Capture Condition Entry

When a capture condition entry is deleted from the proposed system, the capture module deletes corresponding capture flow entries at the OpenFlow switch by sending Flow-Mod messages.

### 3.3. Monitoring Module

The monitoring module is used for traffic monitoring. The behavior of monitoring module is similar to that of capture module. To maintain configuration for traffic monitoring, the monitoring module refers to the monitoring condition table as shown in Figure 3. Each monitoring condition entry in the monitoring condition table consists of condition ID, Datapath ID, conditions, and interval. The interval indicates measurement interval of traffic monitoring. In addition, the traffic monitoring module refers to the flow table. In the following, we explain the details of behavior of monitoring module.

#### 3.3.1. Behavior against Addition of Monitoring Condition Entry

When a monitoring condition entry is added, the monitoring module sends a Flow-Mod message to the corresponding OpenFlow switches. The procedure is same as that in the generation process of basic capture flow entry. In the message, match field is set to the conditions in the monitoring condition entry, the instruction is set to Forward CONTROLLER, and the priority is set to the lowest value 1. We denote the added flow entry for traffic monitoring at the OpenFlow switch as* monitoring flow entry* and the monitoring flow entry with the lowest priority value as* basic monitoring flow entry*.

If there is intersection between a match field in an existing flow entry and conditions in the monitoring condition entry, the monitoring module acts as follows.

- If a match field in an existing flow entry is a part of conditions in the monitoring condition entry, the monitoring module selects the flow entry as monitoring flow entry. We denote the monitoring flow entry as* piggyback monitoring flow entry*.
- On the other hand, if a part of match field in an existing flow entry matches with a part of conditions in the monitoring condition entry, the monitoring module generates Flow-Mod message. The procedure is same as that in the generation process of additional capture flow entry. In the message, the match field is set to the intersection of the existing match field and conditions in the monitoring condition entry. The instruction is set based on the instruction in the existing flow entry and Forward CONTROLLER instruction. The priority is set based on the existing flow entry by adding one. We denote the monitoring flow entry made by this message as* additional monitoring flow entry*.

#### 3.3.2. Behavior of Traffic Monitoring

The monitoring module sends Flow-Stats Request message for each monitoring flow entry at the OpenFlow switch at the designated interval. When an OpenFlow switch receives the message, it replies Flow-Stats Reply message. The Flow-Stats Reply message contains traffic information related to the flow entry. Therefore, our proposed system can monitor the traffic information at this stage.

#### 3.3.3. Behavior against Reception of Flow-Mod Message

When our proposed system receives a Flow-Mod message from OpenFlow controller, the monitoring module acts depending on the relationship between flow entry in the Flow-Mod message and monitoring condition entry as follows.

- If a match field in the Flow-Mod message is a part of conditions in a monitoring condition entry, the monitoring module forwards the message to the OpenFlow switch. The monitoring flow entry made by this message corresponds to piggyback monitoring flow entry.
- On the other hand, if a part of match field in the Flow-Mod message matches with a part of conditions in a monitoring condition entry, the monitoring module forwards the message to the OpenFlow switch. In addition, the monitoring module generates additional Flow-Mod message. In the additional message, the match field is set to the intersection of the match field in the message and conditions in the monitoring condition. The instruction is set based on the instruction in the flow entry in the message. The priority is set based on the flow entry in the message by adding one. The monitoring flow entry made by this message corresponds to additional monitoring flow entry.

#### 3.3.4. Behavior against Reception of Flow-Removed Message

When a flow entry is deleted at the OpenFlow switch due to some reason such as timeout, Flow-Removed message is sent to our proposed system. If a flow entry related to additional monitoring entry is deleted at the switch, the monitoring module deletes the additional monitoring entry at the OpenFlow switch by sending a Flow-Mod message.

#### 3.3.5. Behavior against Removal of Monitoring Condition Entry

When a monitoring condition entry is deleted from the proposed system, the monitoring module deletes corresponding monitoring flow entries at the OpenFlow switch by sending Flow-Mod messages.

### 3.4. REST API Module

Our proposed system provides REST API for interfaces. Therefore, our proposed system can be used not only by administrator but also by other systems for cooperative control. For example, the OpenFlow controller can use the monitoring results from our proposed system for efficient routing. On the other hand, by analyzing packets, it is possible to block unusual traffic or reroute the traffic to a honey pot. The list of APIs in our proposed system is summarized in Table 3.

## 4. Experimental Evaluations

We implemented our proposed system using Java. Although we do not explain details, we confirmed that our proposed system can capture packets and monitor traffic information depending on configuration.

In this section, we conduct several experiments to demonstrate the feasibility of our proposed system and to evaluate performance of our proposed system. We evaluate our proposed system in terms of overhead, packet capturing capability, and effect on data plane performance. In the following, we explain the evaluation results of our proposed system.

### 4.1. Evaluation of Overhead

We first evaluate the overhead of our proposed system. Since our proposed system acts as a proxy, it affects the OpenFlow network. Therefore, we evaluate the overhead by comparing the performance of OpenFlow network with our proposed system and that without our proposed system.

**Figure 9:**
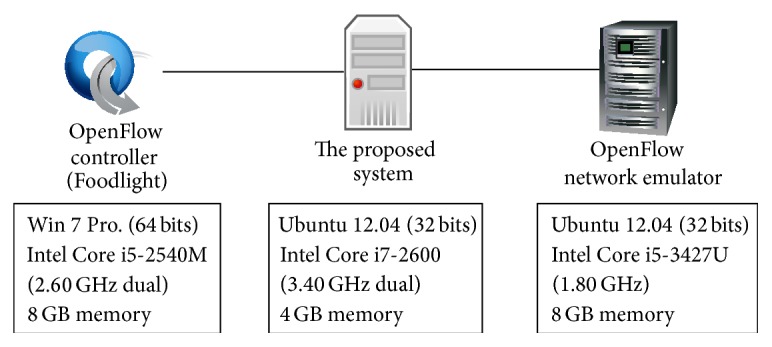
Experimental environment (physical layout) in this paper.

**Figure 10:**
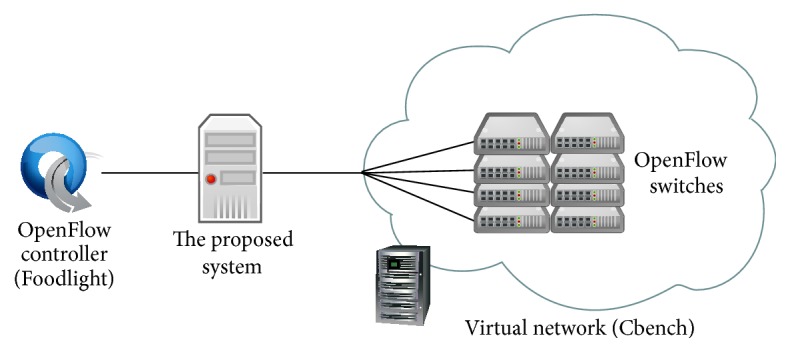
Network topology for evaluating control overhead.

Figure 9 shows the experimental environment in this section. We use Floodlight [17] for OpenFlow controller. In addition, we use Cbench [18], which is a tool to evaluate performance of OpenFlow controller, to emulate virtual OpenFlow switches. The logical network topology of this experiment is shown in Figure 10. In the experiment, we place *n* virtual OpenFlow switches, each of which is connected to 1000 virtual hosts with different MAC addresses. Each virtual OpenFlow switch sends 10000 Packet-In messages to the OpenFlow controller. When the OpenFlow controller receives a Packet-In message, it replies a Flow-Mod message to the virtual OpenFlow switch. As performance metrics, we use the number of responses, which is the total number of Flow-Mod messages received at the virtual OpenFlow switches per a unit time. The conditions in the capture condition entry and that in the monitoring condition entry are set to match all packets. Therefore, all packets are captured and monitored at our proposed system. The results are averaged over 10 experiments.

**Figure 11:**
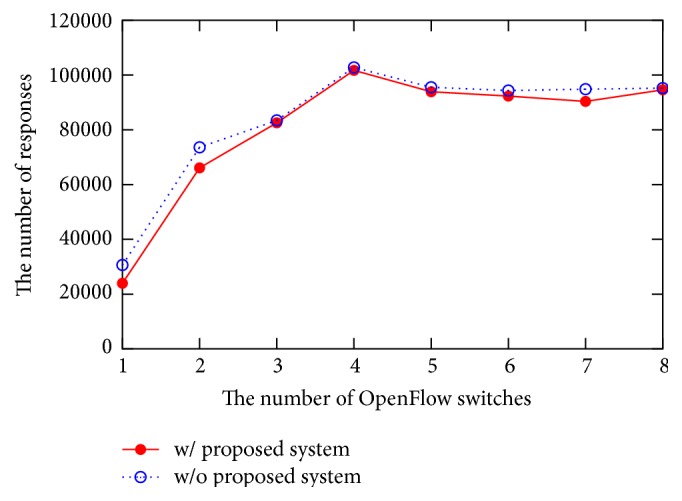
Evaluation on overhead.

Figure 11 shows the number of responses against the number of virtual OpenFlow switches. For comparison, we also show the results without our proposed system. In this case, OpenFlow switches are directly connected to the OpenFlow controller. As shown in Figure 11, both results are similar. It indicates that our proposed system does not influence significant degradation to the performance between controller and switches.

### 4.2. Evaluation of Packet Capturing Capability

Next, we evaluate the performance of proposed system. We note here that our proposed system consists of two main modules: capture module and monitoring module. Since the monitoring module only sends control packets among our proposed system and OpenFlow switches, the overhead is comparatively small. On the other hand, the overhead of capture module is comparatively high since it sends not only control packets but also data packets among our proposed system and OpenFlow switches. Therefore, we evaluate the performance of capture module as the performance of proposed system in this paper because performance evaluation of capture module is more important compared to evaluation of monitoring module.

**Figure 12:**
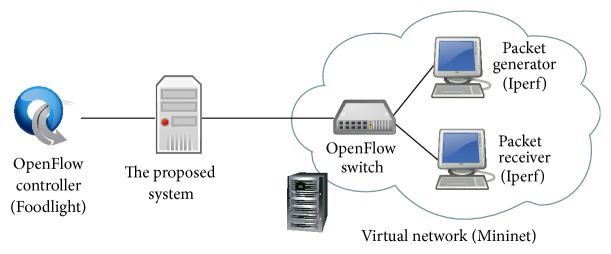
Network topology for evaluating packet capture performance.

We use same physical layout from previous experiments as shown in Figure 9. To emulate an OpenFlow switch and hosts, we use Mininet [19]. The logical network topology of this experiment is shown in Figure 12. In the experiment, a packet generator and a packet receiver are connected to the virtual OpenFlow switch. As a packet generator and receiver, we used Iperf [20]. The virtual OpenFlow switch is connected to the OpenFlow controller through our proposed system. At our proposed system, we add a capture condition entry to capture packets whose source IP address is the packet generator's IP address. We compared the number of received packets at the proposed system and that of the packet receivers.

**Figure 13:**
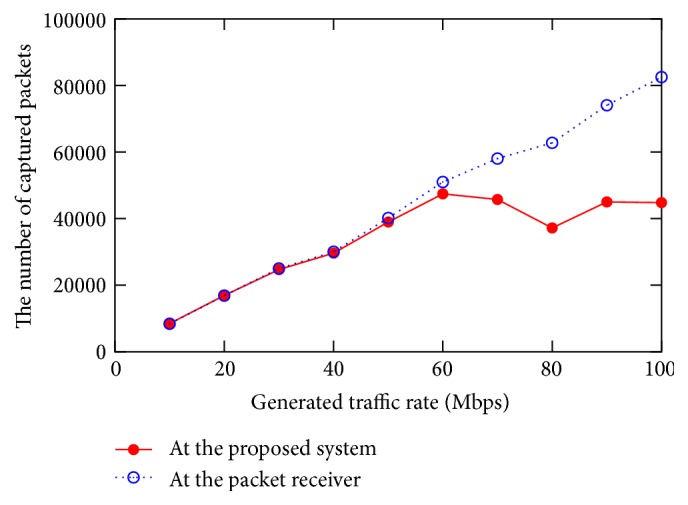
Evaluation on packet capture performance.

Figure 13 shows the number of received packets against generated traffic rate at the packet generator. As shown in Figure 13, the current implementation of our proposed system can capture packets up to 60 Mbps. We plan to improve the packet capture capability at the higher rate condition as future work.

### 4.3. Evaluation on the Effect of Data Plane Performance

Finally, we evaluate the effect of our proposed system on data plane performance. In our proposed system, when an administrator sets conditions, new entries are added to the OpenFlow switches. Therefore, it might affect the performance of lookup of flow entry at the OpenFlow switch. In this experiment, we evaluate the effect of addition of conditions on the performance of data plane. The network topology used in this experiment is same as that in the previous section as shown in Figure 12. In the experiment, we add both capture condition entries and monitoring conditions entries to our proposed system. Therefore, all packets are captured and monitored at our proposed system. We then evaluate the throughput between Iperf server and Iperf client.

**Figure 14:**
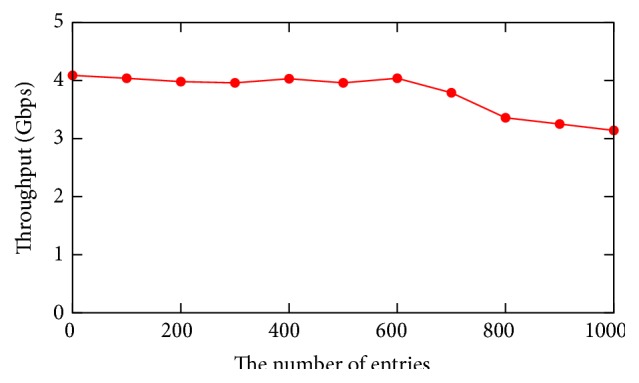
Evaluation on the effect of data plane control.

Figure 14 shows the throughput between Iperf server and client against the number of condition entries. As shown in Figure 14, the number of condition entries does not affect significant degradation of data plane performance. When the number of condition entries is higher than 600, the throughput decreases to the number of entries. However, it is not an important problem since applying 600 condition entries to one OpenFlow switch is not common in actual situations.

## 5. Conclusion

In this paper, we proposed, designed, and implemented a proxy-based network monitoring system for OpenFlow networks. Through experimental evaluations, we confirmed that our proposed system can capture packets and monitor traffic information depending on administrator's configuration. In addition, we showed that our proposed system does not influence significant performance degradation to overall network performance.

As future work, we plan to improve the capture module to enable capturing high rate traffic. In addition, in this paper, we assumed that single monitoring system is installed in an OpenFlow network. As future work, we should take into account cooperation of several monitoring systems for load balancing. Furthermore, the current implementation of our proposed system is based on OpenFlow 1.0. We should extend our implementation to handle OpenFlow 1.1 and later versions.

## Figures and Tables

**Table 1:** Comparison of traditional monitoring systems and our proposed system.

Functions	SNMP [6]	NetFlow [7]	Big tap [8]	Our proposal
Interface-level monitoring	✓	✓	✓	✓
Flow-level monitoring		✓	✓	✓
Packet capture			✓	✓
Vendor independence	✓			✓

**Table 2:** The constitution of conditions for capturing packets.

Layer	Entry
L1	Incoming interface
	Outgoing interface

L2	Source MAC address
	Destination MAC address
	Ethernet type
	VLAN ID

L3	Source IP address
	Destination IP address
	IP version

L4	Source port number
	Destination port number

**Table 3:** List of REST APIs.

URI	Method	Comment
/api/ofswitch/dpid/json	GET	Retrieve a list of Datapath IDs of OpenFlow switches

/api/topology/openflow/links/json	GET	Retrieve a list of links among OpenFlow switches

/api/hosts/json	GET	Retrieve a list of hosts connected to OpenFlow switches

/api/sniffer/rules/json	GET	Retrieve a list of condition entries in the capture condition table
	POST	Set a condition entry to the capture condition table
	DELETE	Delete a condition entry from the capture condition table

/api/traffic/rules/json	GET	Retrieve a list of condition entries in the monitoring condition table
	POST	Set a condition entry to the monitoring condition table
	DELETE	Delete a condition entry from the monitoring condition table

/api/traffic/bytes/id/json	GET	Retrieve the amount of traffic in bytes which matches with an entry in the monitoring condition table

/api/traffic/backets/id/json	GET	Retrieve the number of packets which matches with an entry in the monitoring condition table
